# The Molecular Aspect of Nephrolithiasis Development

**DOI:** 10.3390/cells10081926

**Published:** 2021-07-29

**Authors:** Paulina Wigner, Radosław Grębowski, Michal Bijak, Janusz Szemraj, Joanna Saluk-Bijak

**Affiliations:** 1 Department of General Biochemistry, Faculty of Biology and Environmental Protection, University of Lodz, Pomorska 141/143, 90-236 Lodz, Poland; joanna.saluk@biol.uni.lodz.pl; 2 Department of Urology, Provincial Integrated Hospital in Plock, Medyczna 19, 09-400 Plock, Poland; radek.grebowski@gmail.com; 3 Department of Medical Biochemistry, Medical University of Lodz, Mazowiecka 6/8, 90-001 Lodz, Poland; janusz.szemraj@umed.lodz.pl; 4 Biohazard Prevention Centre, Faculty of Biology and Environmental Protection, University of Lodz, Pomorska 141/143, 90-236 Lodz, Poland; michal.bijak@biol.uni.lodz.pl

**Keywords:** oxidative stress, inflammation, angiogenesis, purine metabolism, urea cycle, nephrolithiasis

## Abstract

Urolithiasis is the third most common urological disease after urinary tract infections and prostate diseases, and it is characterised by an occurrence rate of about 15%, which continues to rise. The increase in the incidence of kidney stones observed in recent decades, is most likely caused by modifications in dietary habits (high content of protein, sodium and sugar diet) and lifestyle (reduced physical activity) in all industrialised countries. Moreover, men are more likely than women to be diagnosed with kidney stones. A growing body of evidence suggests that inflammation, oxidant–antioxidant imbalance, angiogenesis, purine metabolism and urea cycle disorders may play a crucial role in nephrolithiasis development. Patients with urolithiasis were characterised by an increased level of reactive oxygen species (ROS), the products of lipid peroxidation, proinflammatory cytokines as well as proangiogenic factors, compared to controls. Furthermore, it has been shown that deficiency and disorders of enzymes involved in purine metabolism and the urea cycle might be causes of deposit formation. ROS generation suggests that the course of kidney stones might be additionally potentiated by inflammation, purine metabolism and the urea cycle. On the other hand, ROS overproduction may induce activation of angiogenesis, and thus, allows deposit aggregation.

## 1. Introduction

Urolithiasis is the third most common urological disease after urinary tract infections and prostate diseases. A recent epidemiological summary from seven countries, including Italy, Germany, Scotland, Spain, Sweden, Japan, and the United States, revealed incidence rates of nephrolithiasis of 114–720 per 100,000 individuals, and the prevalence rates of 1.7–14.8% are still rising [1]. It has been observed that nephrolithiasis incidence has increased dramatically over the past 30 years, likely due to environmental changes, including inadequate diet and restricted physical activity [2]. According to data from the National Health and Nutrition Examination Survey (NHANES), the self-reported prevalence of kidney stones increased nearly three-fold in the United States from 2007 to 2010 [3,4]. In the United Kingdom, the lifetime prevalence of urolithiasis increased by 63% between 2000 and 2010 [5]. Previous epidemiological studies have showed that the propensity to form stones varies according to sex, ethnicity and geography. Until recently, deposits in the urinary tract were 2–3-times more common in men than in women. However, recent studies show that this disparity between men and women is disappearing, e.g., between 1970 and 2000 in Minnesota (the United States), the male to female ratio of kidney stones incidence decreased from 3.1 to 1.3 [6]. It has been suggested that the reason for the increasing frequency of stones incidence among females might be the changes in lifestyle and diet, resulting in increased obesity among women, which is a known risk factor for deposit formation in the urinary tract [7]. Similarly, the data from Eastern countries, such as China and Japan, where the dietary transition towards “Western diets” has been observed, showed that the nephrolithiasis risk increased [8,9]. “Western diets” are characterised by high consumption of animal protein, which leads to an increase in the excretion of calcium, oxalate, and uric acid in the urine, consequently predisposing to kidney stones. On the other hand, the traditional Chinese diet includes less animal protein and a higher proportion of vegetables than the diet in some Western countries, and thus, might be good for the prevention of deposit formation [8]. An epidemiological study of the male population confirmed a close relationship between changing eating habits and modulating the risk of developing kidney stones in Japan. An increase in BMI (body mass index) was positively correlated with an increased risk of nephrolithiasis [9]. Thus, the risk factors for nephrolithiasis include a high content of protein, sodium (salt) and sugar in the diet, and reduced physical activity, among others. Additionally, diminished fluid and calcium consumption along with increased oxalate consumption might promote kidney stone formation [10,11,12,13,14]. The risk of developing kidney stones is associated not only with the high supply of animal proteins, but also with the presence of certain prohibited substances added by food producers. In September 2008, the melamine contamination of baby milk scandal was exposed. Melamine is a chemical used to increase the nitrogen content of the diluted milk, giving it the appearance of higher protein content to pass quality control tests. The number of victims who consumed the melamine contained in milk has been estimated as 240,000, of which six children died from kidney stones and other kidney damage, and approximately 54,000 children were hospitalised. According to data from the World Health Organization, 82% of the children who were among the victims of melamine consumption were two years of age or below [15,16,17,18]. As mentioned above, stone prevalence is also associated with racial and ethnic differences. A previous analysis confirmed that non-Hispanic white individuals had the highest stone risk (10.3%), followed by Hispanics (6.4%) and non-Hispanic African Americans (4.3%) [3]. Additionally, kidney stone incidence is also associated with environmental factors. Hot, arid climates might contribute to nephrolithiasis development [10]. Previous epidemiological studies carried out in the United States showed that regions with higher average temperatures have the highest risk of urinary tract stone occurrence. Thus, this correlation may suggest that global warming may impact the development of stones [4,11]. Brikowski and colleagues (2006) predicted that, based on the effects of global warming, the percentage of people living in areas designated as high risk for kidney stone formation would increase from 40% in 2000 to 56% by 2050, and up to 70% by 2095 [12]. Moreover, epidemiological data also suggests that stone incidence may have a seasonal character. A higher rate of stone incidence was observed during the warmer summer months than in the colder winter months [13]. Moreover, numerous systemic diseases, including diabetes, metabolic syndrome, cardiovascular disease and obesity are also nephrolithiasis risk factors [14,19,20,21].

Unfortunately, nephrolithiasis is characterised by a high risk of recurrence. The spontaneous 5-year recurrence rate is 35% to 50% following the first renal colic [22]. Moreover, if patients do not apply meta-phylaxis, the relapse rate of secondary stone formation is estimated to be 10–23% per year, 50% in 5–10 years, and 75% in 20 years from the first occurrence [23]. Unfortunately, due to the repeatability of episodes of nephrolithiasis, urinary stones can lead to several complications, which can range from acute to chronic conditions, including pyelonephritis, oilseeds, urinary tract infection, renal insufficiency, and even urinary tract cancer [24,25]. Interestingly, as the incidence of urinary calculi and bladder cancer are all trending upwards, urinary calculi may be involved in bladder cancer development. A meta-analysis confirmed that patients with urolithiasis had an overall 1.87-fold increased risk of bladder cancer as compared with healthy controls. Moreover, Yu et al. (2018) showed that patients with bladder stones showed a higher risk of bladder cancer than patients with kidney stones (2.17 vs. 1.39) [26]. 

Apart from environmental and etiological factors, recent studies also indicate the role of genetic factors in the mechanism of urolithiasis development. A positive family history of stones has been reported in 16% to 37% of patients who have formed kidney stones as compared to 4% to 22% in controls [27]. Epidemiological studies confirm that stone formation occurs at younger ages in patients with a family history of nephrolithiasis. Moreover, patients with a positive family history had relatively more stone episodes from the onset of the disease. The mean time interval between recurrences was noted as significantly shorter in a group of patients with a positive family history when compared with patients without the family history [28]. 

Due to the high prevalence and recurrence of urolithiasis in the world population, it is also associated with high costs of medical care. The total cost of caring for urolithiasis patients, including inpatient, outpatient, and emergency services, was estimated at $2.1 billion in the United States in 2000. Moreover, this cost increased by 50% between 1994 and 2000 [29]. Interestingly, it has been estimated that by 2030 the cost of care for urolithiasis patients will increase in the United States by $1.24 billion per year because of the rising prevalence of obesity and diabetes [30]. 

Despite extensive studies into the pathogenesis of kidney stones, its molecular basis remains unclear. On the other hand, a growing body of evidence suggests that inflammation, oxidant-antioxidant imbalance, purine metabolism, urea cycle as well as angiogenesis disorders may play a significant role in the formation of deposits in the urinary tract. Thus, in this article, we aim to elucidate, based on available literature, the role of the abovementioned biochemical pathways in the pathogenesis of kidney stones.

## 2. Oxidative Stress

**Figure 1:**
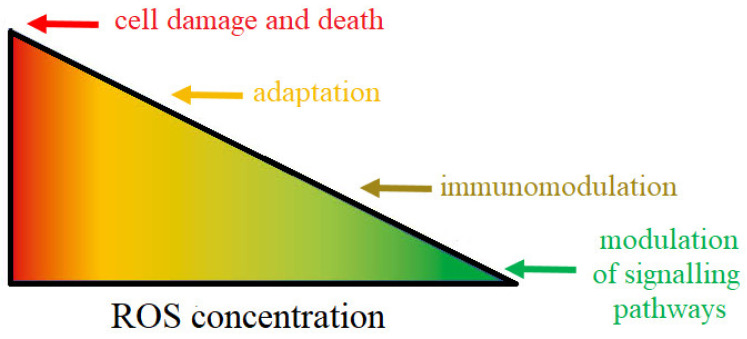
Under conditions of homeostasis, reactive oxygen radicals are released in amounts safe for the cell and can induce cell differentiation and apoptosis, influence the synthesis, release or inactivation of nitric oxide, and stimulate glucose transport to cells. By increasing the permeability of the capillary walls, they ensure the proper course of the inflammatory reaction. One of the most important tasks performed by ROS is the regulation of the processes of signal transmission from cell to cell and within it. Higher concentrations of these molecules cause toxic cell damage, leading to their destruction. The harmful effect of free oxygen radicals is manifested, among others, by their ability to oxidise proteins, nucleic acids and lipids [51].

Reactive oxygen species (ROS) are highly reactive compounds that play a critical role as signalling molecules, but can also cause damage to proteins, lipids, carbohydrates and nucleotides, leading to the development of various diseases, including kidney stones [31,32]. Under conditions of homeostasis, ROS play an important role in regulating the processes of signal transmission from cell to cell and within it. For example, due to the low reactivity, selectivity and constant availability in the cell, among ROS, the superoxide ion and hydrogen peroxide play the role of modulators in the adenylate cyclase and the phospholipase C pathways [31,33]. ROS are also involved in the regulation of receptors’ function, mainly those containing -SH (thiol) groups. Most often, the thiol groups are in ligand binding sites. As a result of the action of ROS, oxidation of the thiol groups takes place, which leads to inactivation of the ligand binding site, and thus, the entire receptor. On the other hand, ROS can enhance the activity of transport proteins, such as 5-lipoxygenase, which is an additional source of free radicals generated by stimulated lymphocytes [34,35]. Another important activity of ROS is related to the response of phagocytic cells (granulocytes, monocytes, macrophages) to pathogens (oxygen explosion phenomenon) [36]. ROS are also involved in the regulation of immune processes. Previous studies showed that ROS intensify the T lymphocytes activation and induce the adhesion of leukocytes to the endothelium, which allows their penetration from the circulatory system to the site of the inflammatory reaction [31,33,37]. Moreover, low hydrogen peroxide concentration stimulates the activity of the nuclear factor kappa-light-chain-enhancer of activated B cells (NF-κB), which activates the expression of many genes, including cytokines (interleukin-IL-1β or IL-6), thioredoxins, SOD (superoxide dismutase) [38]. ROS also affect cell survival, ageing and death. Low concentrations of ROS regulate the processes of cell differentiation and allow them to adapt to changed conditions, while exposure to higher concentrations of free radicals induces apoptosis, which allows the elimination of those cells that are heavily damaged and could pose a risk to the body [33,39]. As mentioned above, high concentrations of ROS are dangerous to cells. The harmful effect of free oxygen radicals is manifested, among others, by their ability to oxidise proteins, nucleic acids and lipids [33,40,41]. Protein oxidation can lead to a break in the polypeptide chain, the appearance of altered residues’ amino acids and the formation of protein dimers or aggregates. Thus, these changes result in the loss of the functional activity of enzymes, regulatory proteins or membrane transporters [42,43,44]. Proteins that have undergone irreversible changes are selectively removed by proteases, but as cells age and their proteolytic activity is lowered, they can accumulate in the cell, leading to its permanent damage. ROS, including the hydroxyl radical and oxygen singlet, can also damage purine and pyrimidine bases, residues’ sugar or lead to breaking of the phosphodiester bonds connecting nucleotides [40,41,45]. As a result of ROS activity, nucleic acid strand breaks or products of their oxidative modification, including 8-hydroxy-2-deoxyguanine and 8-hydroxyguanine, can occur in mitochondria and cell nuclei. Due to its proximity to the mitochondrial respiratory chain, mitochondrial DNA is more susceptible to oxidative damage than nuclear DNA [41,46,47]. Lipids, especially polyunsaturated fatty acids, are also particularly exposed to high concentrations of ROS. As a result of lipid peroxidation, modified, damaged lipid molecules forms. Among the peroxidation products of polyunsaturated fatty acids, malondialdehyde or 4-hydroxynonenal are the most common [48]. Additionally, lipid peroxidation products, such as 4-hydroxynonenal, can adversely affect protein activity. 4-hydroxynonenal related proteins (e.g., glutathione transferase) change conformationally and functionally [49]. In addition, lipid peroxidation products also affect the physical properties of cell membranes, causing, among others, inhibition of the activity of membrane enzymes and transport proteins. Moreover, they can induce the expression of cyclooxygenase type 2 (COX-2) in macrophages and activate the inflammatory potential of these cells [50]. However, cells have developed defence mechanisms against the overproduction of ROS. These systems include enzymes, degrading ROS, and non-enzymatic low-molecular compounds, antioxidants, which, under the action of reactive oxygen, constitute a defence shield for molecules important for the cell [51]. The two faces of ROS are shown in Figure 1.

The primary source of ROS in kidneys is NADPH oxidase, particularly in the presence of angiotensin [52]. Crystal aggregation and retention, crucial events for kidney stones formation, are closely associated with free radical activity in vivo. The deposition of calcium oxalate (CaOx) crystal (the most commonly diagnosed form of urinary tract stones) leads to renin upregulation and angiotensin II production, which consequently cause NADPH oxidase activation, and thereby, intensification of ROS generation. Then, ROS causes activation of transcription factors through P38 mitogen-activated protein kinase (-MAPK)/JNK, including nuclear factor kappa-light-chain-enhancer of activated B cells (NF-κB), activated protein-1 (AP-1) and growth factors including TGFβ Runt-related transcription factor-2 (RUNX-2) and osterix. In consequence, the activation of ROS-induced transcription causes the generation of isoprostanes and prostaglandins, and thereby, may modulate inflammatory response (Khan). ROS-induced NF-kB may also regulate the expression of genes encoding adhesion molecules, COX-2, and pro-inflammatory cytokines, tumour necrosis factor-alpha (TNF-α), interleukin 6 (IL-6), and C-reactive protein (CRP). Then, these factors in a vicious circle mechanism may additionally activate NADPH oxidase and may stimulate ROS generation, which may impair endothelial function [53]. 

Besides NADPH oxidase, mitochondrial production of ROS is the next culprit behind renal injury associated with kidney deposit formation in the pathophysiology of nephrolithiasis. After the heart, the kidney is characterised by the second-highest mitochondrial content and oxygen consumption, and thus, is considered as one of the most human energy-consuming organs. This high energy demand is necessary for proper kidney function, including removal of waste from the blood, reabsorption of nutrients, maintenance of acid-base homeostasis, blood pressure regulation, and maintaining the balance of electrolytes and fluid. However, not every element of the kidney structure has the same energy demand. The kidney structures with the highest energy requirements are the proximal tubules, which reabsorb 80% of the filtrate passing through the glomeruli. Therefore, they require additional active transport mechanisms compared to other types of kidney cells, thus, they have more mitochondria than any other structure in the kidney. Consequently, the proper functioning of the proximal tubule depends on the ability of mitochondria to sense and respond to changes in nutrient availability and energy demand [54,55,56,57,58]. As noted in the introduction, CaOx is the most commonly diagnosed, which is mainly the result of an increased concentration of oxalate in the urine. Previous studies have showed that high oxalate levels cause renal oxidative stress, which could play a crucial role in the initiation and progression of renal cell injury [59,60]. The in vitro and in vivo studies suggest that oxalate may disturb the electron transport chain in mitochondria, and thus, may lead to the leak of free radicals [61]. A subsequent study showed that calcium oxalate monohydrate crystals might inhibit the mitochondrial respiratory chain in proximal tubular cells [62]. It has been shown that as a result of exposure to oxalates, mitochondria increased production of ROS, lipid peroxides and oxidised thiol proteins [63]. The deposition of CaOx crystal may also cause damage to mitochondria through increased cellular ceramide level. The consequences of mitochondria damage via ceramides may be increased hydrogen peroxide production, glutathione depletion and reduction in mitochondrial membrane potential, which results in activation of caspases, and finally, apoptosis induction [64]. Moreover, Niimi and colleagues (2014) suggested that mitochondrial injury may be induced by the opening of mitochondrial permeability transition pores (mPTP) in the inner mitochondrial membrane [65]. The mPTP opening may be a result of calcium overload and oxidative stress. Consequently, the mPTP opening may cause uncoupling of oxidative phosphorylation and compromise the intracellular ATP level, finally leading to necrotic cell death [65].

Thus, pro-oxidant and antioxidant imbalance may contribute to the development of kidney stones. Consequently, patients with kidney stones were characterised by increased lipid peroxides, while nitrite and α-tocopherol levels were reduced in the serum [66]. Previous studies proved that patients with nephrolithiasis were characterised by an increased level of pro-oxidative damage markers in urine, including 8-hydroxydeoxyguanosine (8-oxoG, a marker of DNA oxidative damage) and thiobarbituric acid-reactive substances (TBARS, a marker of lipid peroxidation) [67]. Moreover, an animal study showed that nephrolithiasis may be associated with a decrease in epithelial nitric oxide synthetase (*eNOS*) expression and an increase in inducible nitric oxide synthetase (*iNOS*) expression in the renal medulla of the ethylene glycol (EG)-treated rat, which is known as the established model for CaOx formation in kidneys. Moreover, the level of nitrotyrosine (tyrosine nitration product mediated by reactive nitrogen species) was increased in both the renal cortex and the medulla of EG-treated rat [68]. The reduced mRNA expression of *eNOS* may be a result of changes in the osteopontin (OPN) activity. OPN may downregulate the expression of nitric oxide synthase (*NOS*) in human kidney proximal tubule epithelial cells [69]. Previous results showed that OPN directs calcium oxalate crystallisation to the calcium oxalate dihydrate phase, which is less adherent to the renal tubular epithelial cells than the calcium oxalate monohydrate phase [70]. Thus, ROS exposition was associated with a decreased *NOS* mRNA level and simultaneously with an increased *OPN* mRNA level [69]. Moreover, Khan et al. (2002) found that increased *OPN* expression in kidneys was correlated with increased urinary OPN excretion after the deposition of CaOx in the kidneys [71].

On the other hand, kidney stone formation may be correlated with decreased antioxidant levels, including *α*-carotene, *β*-carotene and *β*-cryptoxanthin [72]. Additionally, in a paediatric population, results of a study confirmed that kidney stones were associated with oxidative stress. Urine total antioxidant status (TAS) and total oxidant status (TOS) were higher in the paediatric patients than controls [73]. The long-lasting oxidative condition may cause an increase in antioxidant levels, and therefore high TAS values may indicate persistent oxidative stress. Moreover, a reduction in antioxidant enzyme activity, including superoxide dismutase (SOD, −7%), glutathione peroxidase (Gpx, −20%) and glutathione-S-transferase (GST, −13%), was also observed in patients with kidney stones. Thus, the decreased antioxidant enzyme activity exacerbates the aforementioned disorders due to the organism’s inability to counter the damaging effects of ROS [74]. On the other hand, surgical removal of the renal calculus may contribute to a decrease in malonyl dialdehyde (MDA, 29%) and an increase in Gpx activity (by 50%), reaching values close to the levels of the control group [74]. Previous studies have suggested that an elevated peroxidation and depletion of thiol level may contribute to an increase in the oxalate binding activity and damage to the renal tubular cells, which in turn promotes nucleation, crystal adherence and aggregation of stones [66]. Therefore, in idiopathic CaOx stone cases, a high urine level of renal enzymes indicative of renal tubular damage was observed, such as γ-glutamyl transpeptidase, angiotensin 1 converting enzyme, β-galactosidase and N-acetyl-beta-D-glucosaminidase (NAG), and products of lipid peroxidation, including MDA. Moreover, urinary NAG excretion was correlated positively with 8-OHdG concentration [67,75]. Therefore, renal tubular damage is closely associated with ROS generation. 

Interestingly, *MnSOD* (manganese superoxide dismutase) polymorphism may be used as a tool to identify individuals who are at risk of urolithiasis. A previous study showed that the Ala/Ala genotype of Ala-9-Val *MnSOD* polymorphism decreased the risk, while the Val/Val genotype was associated with increased risk of kidney stones [76]. 

A summary of the pro-oxidant and antioxidant imbalance factors associated with kidney stones has been presented in Table 1.

## 3. Inflammation

As previously noted, ROS overproduction can lead to the development of inflammation. On the other hand, inflammation may also be directly associated with stone formation. In the course of urolithiasis, it has been observed that the CaOx crystals can be retained in the kidneys via binding to the tubular cells and then aggregating to large forms [77]. Thus, the renal deposits can stimulate renal cells to secrete inflammatory mediators, such as monocyte chemoattractant protein-1 (MCP-1, also known as CCL2) and TNF-α [78,79]. Previous studies showed that renal cell injury and inflammation relating to the development of renal stones were associated with an increase in IL-6 in the urine of patients with urolithiasis [80,81,82]. Moreover, a renal biopsy from kidneys containing calculi showed increased mRNA expression of *IL-6* and *MCP-1* in kidney tissue [83]. Additionally, Hasna and colleagues (2015) found that diabetic patients with urolithiasis were characterised by higher CRP and IL-6 levels than diabetes mellitus cases without urolithiasis [84]. The increased level of serum CRP correlated with self-reported kidney stone incidences in younger individuals [85].

**Figure 2:**
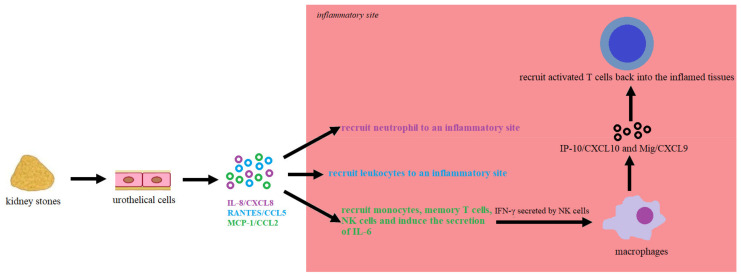
Initiation of “chemokine to cytokine to chemokine” cascade. Kidney stones may contain appreciable amounts of endotoxin, and can stimulate tubular epithelial to secrete chemokines, including IL-8/CXCL8, RANTES/CCL5 and MCP-1/CCL2, via TLR4. IL-8/CXCL8 plays a key role in the recruitment of neutrophils, while RANTES/CCL5 is known for leukocyte recruitment to an inflammatory site. In turn, MCP-1/CCL2 recruits monocytes, memory T cells, NK cells, and induces IL-6 and expresses intercellular adhesion molecule-1 secretion by tubular epithelial cells. Moreover, activated NK cells release IFN-γ, which can stimulate resident tissue cells to produce IP-10/CXCL10 and Mig/CXCL9, which are ultimately responsible for guiding activated T cells back into the inflamed tissues. Thus, stone-induced renal damage can initiate a “chemokine to cytokine to chemokine” cascade, which may play a crucial role in urolithiasis pathogenesis [86,87,88,89,90,91,92,93].

Suen et al. (2010) found that patients with kidney stones were characterised by an increased level of inflammatory biomarkers in urine, including IL-6, IL-8/CXCL8 (interleukin 8/chemokine (C-X-C motif) ligand 8), RANTES/CCL5 (regulated on activation, normal T-cell expressed and secreted/C-C motif chemokine ligand 5), MCP-1/CCL2, Mig/CXCL9 (monokine induced by gamma/chemokine (C-X-C motif) ligand 9) and IP-10/CXCL10 (C-X-C motif chemokine ligand 10/interferon gamma-induced protein), the most sensitive of which turned out to be IL-8/CXCL8. Moreover, patients with recurrent stones had higher urinary IL-8 levels than patients with the first episode of renal colic [86]. The renal stones can stimulate tubular epithelial and immune cells to secrete inducible chemokines via TLR4 (Toll-like receptor 4) and TLR2 (Toll-like receptor 2), including IL-8/CXCL8, RANTES/CCL5 and MCP-1/CCL2 [87,88]. In response to agonists of TLR2 and TLR4, dendritic cells can produce RANTES/CCL5, IP-10/CXCL10, IL-8/CXCL8, MIP-1α/CCL3 and MIP-1β/CCL4 [89]. In turn, IFN-γ secreted by NK cells can stimulate resident tissue cells to produce IP-10/CXCL10 and Mig/CXCL9. Then, IP-10/CXCL10 and Mig/CXCL9 may guide activated T cells back into the inflamed tissues [90]. The early production of these chemokines from tubular epithelial cells and dendritic cells is essential in shaping the immune response in the kidney [91]. IL-8/CXCL8 may also recruit neutrophil to an inflammatory site, while MCP-1/CCL2 may recruit monocytes, memory T cells and NK cells [92]. Moreover, MCP-1/CCL2 can induce the secretion of IL-6 by tubular epithelial cells and express intercellular adhesion molecule-1 [93]. Therefore, renal damage caused by deposits may initiate a “chemokine to cytokine to chemokine” cascade (Figure 2). Additionally, macrophage inflammatory protein 1β (CCL4, MIP-1β) and interleukin 13 (IL-13) levels were higher in the urine of stone-forming adolescents than controls. The MIP-1 family is involved in chemokine receptor activation, including CCR1 (C-C chemokine receptor type 1) and CCR5 (C-C chemokine receptor type 5), regulating acute and chronic inflammation [94]. In turn, IL-13 may block the production of initiating inflammatory cytokines by monocyte, including IL-6, and increase major histocompatibility complex (MHC) class II expression [95].

Previous polymorphism analysis has showed that the frequency of the T/T (−511) genotype of the *IL-1β* promoter region and the I/II (410/240) genotype of *IL*-*1RA* was higher in kidney stone patients than in control subjects [96]. Similarly, in the Turkish population, polymorphism analysis suggested that IL-1RN and IL-1β (−511) SNPs were associated with urolithiasis [97]. Chen et al. (2001) showed that polymorphism located in IL-1 was associated with the development and severity of adult urolithiasis [98]. On the other hand, Xiao et al. found no significant differences in the occurrence of the genotypes of IL-1RN, IL-1β (−511) and IL-1β (+3954) polymorphism in Uighur children of China [99]. The next polymorphism is located in the IL-18 gene. A/C + C/C genotypes of +105A/C IL-18 polymorphism increased the urolithiasis risk [100]. Similarly, the C/C genotype of the same polymorphism was associated with high nephrolithiasis risk in Iraqi populations [101].

A summary of the inflammatory mediators associated with kidney stones development is presented in Table 2. 

## 4. Purine Metabolism

**Figure 3:**
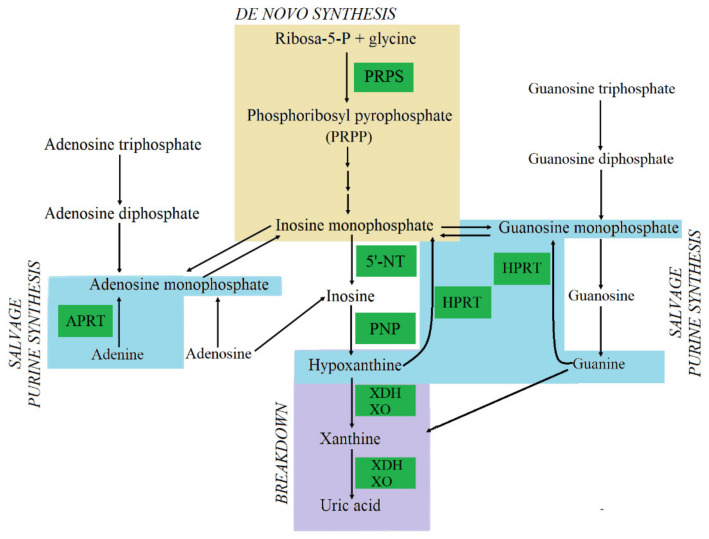
Purine metabolism includes three steps: de novo synthesis, salvage and breakdown pathways. The de novo synthesis is a multi-step process and requires the contribution of PRPS, four amino acids, one PRPP, two folates and three ATP to synthesise an inosine monophosphate (IMP). IMP may be directly catalysed into inosine by 5′-nucleotidases (5′NT), and then purine nucleoside phosphorylase (PNP) converts inosine into hypoxanthine. HPRT catalyses the salvage synthesis of inosine monophosphate (IMP) and guanosine monophosphate (GMP) from the purine bases hypoxanthine and guanine respectively, utilising PRPP as a co-substrate. The HPRT action causes the accumulation of hypoxanthine and guanine, which are converted into uric acid by XO and xanthine dehydrogenase (XDH) in the breakdown pathway. Additionally, APRT catalyses the salvage synthesis of adenosine monophosphate (AMP) and IMP adenine, utilising PRPP as a co-substrate [106].

Another metabolic pathway associated with stone formation is purine metabolism (Figure 3). Previous studies have confirmed that the deficiency of enzymes involved in purine metabolism may be a cause of kidney stone development. One of them is adenine phosphoribosyltransferase (APRT) deficiency, leading to increased conversion of adenine (which it normally converts into AMP) into 2,8-dihydroxyadenine (2,8-DHA), which is 50 times less soluble than uric acid and leads to renal stone formation [102]. Similarly, hypoxanthine-guanine phosphoribosyltransferase (HPRT) deficiency, known as Lesch–Nyhan syndrome, is an X-linked disorder that causes urinary tract deposit formation. The syndrome is associated with the increased degradation of guanine and hypoxanthine to uric acid [103]. A high amount of uric acid in the urine leads to uric acid crystals forming. On the other hand, xanthine oxidase (XO) deficiency also leads to kidney stone formation, since xanthine and hypoxanthine cannot metabolise into uric acid, and thus it has been shown to increase urine xanthine and hypoxanthine concentration [104]. Xanthine is the least soluble of all purines excreted in the urine and increased urinary xanthine level can lead to precipitation and stone formation. Thus, the plasma level of uric acid and oxy-purines reflects disorders of purine metabolism. Hypouricemic patients are characterised by a low level of uric acid below 80 µmol/L (normal level is 300–360 µmol/L) and increased plasma concentrations of xanthine and hypoxanthine, 7 to 20 times and 1.5 to 3.5 times more than normal, respectively [103]. Previous results also indicate that phosphoribosylpyrophosphate synthetase (PRPS) super activity may lead to the development of kidney or bladder stones that may result in episodes of acute kidney failure [105].

A summary of purine metabolism enzymes associated with kidney stone development is presented in Table 3.

## 5. Urea Cycle and Polyamine Biosynthesis

**Figure 4:**
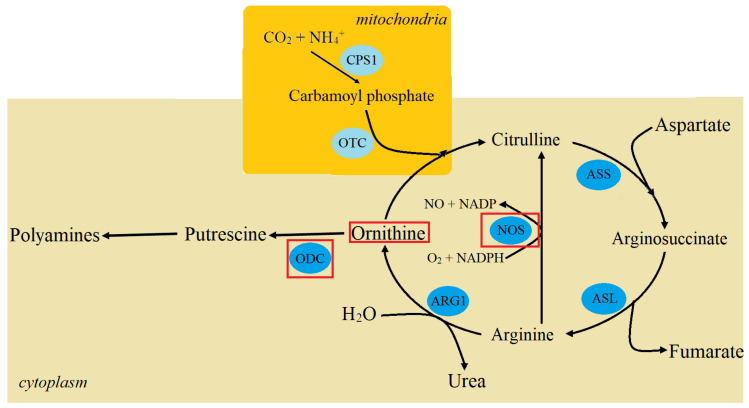
The urea cycle includes five reactions localised at mitochondria of hepatocytes and cytoplasm. The first step involves the CO and ammonia conversion into carbamoyl phosphate via carbamoyl phosphate synthetase I (CPS I). Carbamoyl phosphate and ornithine (whose elevated plasma level and decreased urine level are observed in patients with nephrolithiasis) combine to citrulline via ornithine transcarbamoylase (OTC). Then ornithine translocase transports citrulline from hepatocyte mitochondria into the cytoplasm. Citrulline reacts with aspartate and argininosuccinate forms. This reaction is catalysed by argininosuccinate synthetase (ASS). Argininosuccinate is converted into arginine via argininosuccinate lyase (ASL). Finally, arginase (ARG1) hydrolyses arginine into urea and ornithine. On the other hand, L-arginine may be oxidatively degraded by NOS into L-citrulline and nitric oxide (NO). In the course of nephrolithiasis, a decrease in *eNOS* expression has been observed. Moreover, ornithine from the urea cycle may be converted by ornithine decarboxylase (ODC) in the polyamine biosynthetic pathway. *ODC* polymorphism may be associated with an increased risk of kidney stones [115].

The urea cycle (Figure 4) includes crucial pathways involved in urolithiasis progression. The regulation of the Krebs cycle in kidneys may contribute to stone formation by modifying microenvironmental pH and the induction of ROS generation [107]. Patients with kidney stones are characterised by higher plasma amino acids levels than controls, including taurine, aspartic acid, hydroxyproline, glutamic acid, glycine, alanine, cystathionine, ornithine and lysine [108]. On the other hand, decreased urine ornithine and tyrosine levels were observed in the urine of CaOx patients. L-ornithine is a crucial amino acid involved in the urea cycle and polyamine biosynthesis [109,110]. Ornithine transformation is regulated by ornithine decarboxylase (ODC) [111]. Previous studies showed that *ODC* expression might be modulated by the polymorphism located at the *ODC* gene intron 1. at position +316 [112]. This SNP is located between two c-myc transcription factor binding sites, and a rare allele (allele A) leading to a decrease in the *ODC* gene expression [113]. However, +316 G/A polymorphism of *ODC* cannot serve as a genetic biomarker of stone disease. The frequency of SNP genotypes was similar in urolithiasis and control groups [114]. Moreover, in the course of urolithiasis, changes in *NOS* expression have been observed, which was discussed above in Section 2 on oxidative stress.

## 6. Angiogenesis Genes

Interestingly, disorders of angiogenesis might be crucial to nephrolithiasis development. Crystal adhesion is one of the key steps in kidney deposit formation. The cellular matrix, which is located nearest to the stone and mainly consists of hyaluronic acid (HA), interacts with various intracellular and extracellular molecules via cell surface receptors, including CD44 and CD168. In turn, CD44 has an affinity towards many other ligands, such as osteopontin, collagen, and matrix metalloproteinases (MMPs). These components of the extracellular matrix (ECM) are potent crystal binding molecules that are involved in adherence and internalisation of kidney stone crystals, causing aggregation, growth, and, finally, stone formation [116,117]. Additionally, increased MMPs activation may be induced by ROS [118,119]. High ROS level leads to an increase in transcription factor 1 (AP-1) levels and increased nuclear translocation of NF-kB, causing increased *MMPs* expression, including *MMP*-*9* [120]. In turn, an increased level of MMPs intensifies the processes of ECM remodelling. Additionally, the cleavage products of collagen and osteopontin degradation by MMPs (specifically MMP-9) stimulate a chemotactic response and contribute to inflammatory cell recruitment, which is also associated with kidney stone development [121]. MMP-9 stimulates the migration of dendritic cells and induces cleavage of E-cadherin surface receptor, CD103, on dendritic cells, facilitating the epithelial-mesenchymal transition, and thus allows deposit adhesion and aggregation [122].

Previous studies of the role of angiogenesis in nephrolithiasis development have focused primarily on the analysis of the *MMP*-*9* polymorphisms. The homozygote T/T of the C1562T polymorphism of *MMP*-*9* promoter region (rs3918242) was more frequent in nephrolithiasis patients than controls. Moreover, nephrolithiasis patients with C/T and T/T genotypes were characterised by increased serum MMP-9 levels, TOS, MDA, and uric acid AS compared to nephrolithiasis patients with the C/C genotype. Thus T/T genotype of the C1562T polymorphism in the *MMP*-*9* was associated with an increased risk of stones and intensification of oxidative stress processes [123]. Moreover, the polymorphism located in the 3′-untranslated region of the *MMP*-*9* (rs1056628) was associated with susceptibility to idiopathic calcium nephrolithiasis in the Chinese population. The C/C genotype of the SNP increased the risk of kidney stones. Moreover, A→C transition of this SNP in the miR-491-5p recognition site of *MMP9* enhanced gene expression, potentially through the inhibition of miR-491-5p binding. Thus, mRNA and protein MMP9 levels were higher in nephrolithiasis patients with the C/C genotype than those with the A/A genotype [124]. The next polymorphism associated with modulation of the risk of kidney stones is Bst U I C/T. This polymorphism of the *VEGF* gene is located upstream at the -460th nucleotide. The T allele of this *VEGF* polymorphism was more common in the group of patients with stone diseases than controls [125]. Therefore, the studied SNPs may be used as potential biomarkers to predict the development of nephrolithiasis.

## 7. Conclusions

In summary, the development of kidney stones is the result of disturbances in the course of related biochemical pathways, such as oxidative stress, inflammation, purine metabolism, the urea cycle and angiogenesis. Our review allows us to identify the common factors involved in nephrolithiasis pathomechanisms for the analysed pathways. On the one hand, proinflammatory cytokines, disorders of the urea cycle and purine metabolism contributed to the intensification of ROS generation. On the other hand, ROS overproduction may induce the activity of proinflammatory, including IL-6 and TNF-α, and pro-angiogenesis factors. Thus, oxidative stress may allow maintenance of the inflammatory microenvironment, ECM remodelling and finally lead to an aggregation of deposits. However, further research is needed to clarify which factors in the analysed pathways are directly involved in the development of urolithiasis.

## Figures and Tables

**Table 1:** Potential biomarkers in kidney stone diagnostics.

Compound (Abbreviation)	Physiological Role	Disfunction in the Nephrolithiasis Course	Biological Samples	Citations
α-tocopherol	A type of vitamin E that has antioxidant capacity to reduce lipid peroxidation.	Reduced level	Serum	[66]
8-hydroxydeoxyguanosine (8-oxoG)	An oxidised derivative of deoxyguanosine and is one of the major products of DNA oxidation.	Increased level	Urine	[67]
Nitrotyrosine	Tyrosine nitration product mediated by reactive nitrogen species, including peroxynitrite anion and nitrogen dioxide.	Increase level	Renal cortex and medulla imply	[68]
Epithelial nitric oxide synthetase (eNOS) and inducible nitric oxide synthetase (iNOS)	NOSs are a family of enzymes that catalyses the production of nitric oxide from L-arginine. iNOS is in the cytosol, while eNOS is membrane-associated.	Decrease in epithelial eNOS expression and increase in iNOS expression	Renal medulla	[68]
*α*-carotene, *β*-carotene and *β*-cryptoxanthin	These are common carotenoids that form retinol and its antioxidant capacity to reduce lipid peroxidation.	Reduced level	Serum	[72]
Superoxide dismutase (SOD)	An enzyme catalysing the dismutation of the superoxide radical into ordinary molecular oxygen and hydrogen peroxide.	Reduced activity	Serum	[74]
Glutathione-S-transferase (GST)	An enzyme catalysing the conjugation of the reduced form of glutathione (GSH) to xenobiotic substrates for the purpose of detoxification.	Reduced activity	Serum	[74]
Glutathione peroxidase (Gpx)	An enzyme that protects the organism from oxidative damage. It reduces lipid hydroperoxides to their corresponding alcohols as well as free hydrogen peroxide to water.	Reduced activity	Serum	[74]
Malonyl dialdehyde (MDA)	A marker of lipid peroxidation of polyunsaturated fatty acids, including arachidonic acid.	Increased level	Urine	[74]

**Table 2:** Potential biomarkers associated with inflammation in kidney stone diagnostics.

Mediator (Abbreviations)	Physiological Role	Disfunction in the Nephrolithiasis Course	Biological Samples	Citations
Interleukin 6 (IL-6)	IL-6 is a potent inducer of the acute phase response. Rapid production of IL-6 contributes to host defence during infection and tissue injury, but excessive IL-6 synthesis is involved in disease pathology. In the innate immune response, it is synthesised by myeloid cells, such as macrophages and dendritic cells, upon recognition of pathogens through toll-like receptors (TLRs) at the site of infection or tissue injury (probable). In the adaptive immune response, it is required for the differentiation of B cells into immunoglobulin-secreting cells. It plays a major role in the differentiation of CD4+ T cell subsets, is an essential factor for the development of T follicular helper (Tfh) cells that are required for the induction of germinal-centre formation, is required to drive naive CD4+ T cells to the Th17 lineage. It is also required for proliferation of myeloma cells and the survival of plasma blast cells.	Increase level	Urine	[80,81,82,86]
		Elevated mRNA expression	Kidney tissue	[83]
Monocyte chemoattractant protein 1/chemokine (C-C motif) ligand 2 (MCP-1/CCL2)	MCP-1, known as CCL2, is one of the key chemokines that regulate migration and infiltration of monocytes/macrophages.	Elevated mRNA expression	Kidney tissue	[83]
		Increased level	Urine	[86]
C-reactive protein (CRP)	An acute-phase protein of hepatic origin that increases following interleukin-6 secretion by macrophages and T cells. Its physiological role is to bind to lysophosphatidylcholine expressed on the surface of dead or dying cells.	Increased level	Serum	[85]
Interleukin 8/chemokine (C-X-C motif) ligand 8 (IL-8/CXCL8)	IL-8, known also as CXCL8, is a chemokine produced by macrophages, epithelial cells, airway smooth muscle cells and endothelial cells. It induces chemotaxis in neutrophils and other granulocytes, causing them to migrate toward the site of infection. IL-8 also stimulates phagocytosis once they have arrived. IL-8 is also known to be a potent promoter of angiogenesis.	Increased level	Urine	[86]
Regulated on activation, normal T-cell expressed and secreted/C-C motif chemokine ligand 5 (RANTES/CCL5)	CCL5, known also as RANTES, is chemotactic for T cells, eosinophils, and basophils, and plays an active role in recruiting leukocytes into inflammatory sites. With the help of particular cytokines (i.e., IL-2 and IFN-γ) that are released by T cells, CCL5 also induces the proliferation and activation of certain natural-killer (NK) cells to its activated form.	Increased level	Urine	[86]
Monokine induced by gamma/chemokine (C-X-C motif) ligand 9 (Mig/CXCL9)	Mig, also known as CXCL9, plays a role in the induction of chemotaxis, promotes differentiation and multiplication of leukocytes, and causes tissue extravasation.	Increased level	Urine	[86]
C-X-C motif chemokine ligand 10/interferon gamma-induced protein (IP-10/CXCL10)	CXCL10, also known as IP-10, is secreted by monocytes, endothelial cells and fibroblasts in response to IFN-γ. CXCL10 is involved in the chemoattraction for monocytes/macrophages, T cells, NK cells, and dendritic cells, promotion of T cell adhesion to endothelial cells, antitumour activity, and inhibition of bone marrow colony formation and angiogenesis.	Increased level	Urine	[86]
Chemokine (C-C motif) ligands 4/Macrophage inflammatory protein-1β (CCL4/MIP-1β)	CCL4, also known as MIP-1β, is produced by: neutrophils, monocytes, B cells, T cells, fibroblasts, endothelial cells, and epithelial cells. It is a chemoattractant for natural killer cells, monocytes, and a variety of other immune cells.	Increased level	Urine	[94]
Interleukin 13 (IL-13)	IL-13 is a cytokine secreted by T helper type 2 cells, CD4 cells, natural killer T cell, mast cells, basophils, eosinophils. It is a central regulator in IgE synthesis, goblet cell hyperplasia, mucus hypersecretion, airway hyperresponsiveness, fibrosis and chitinase up-regulation as well as a mediator of allergic inflammation and different diseases, including asthma.	Increased level	Urine	[94]

**Table 3:** Potential biomarkers associated with purine metabolism pathway in kidney stone diagnostics.

Factor (Abbreviation)	Physiological Role	Disfunction in the Nephrolithiasis Course	Biological Samples	Citations
Adenine phosphoribosyl transferase (APRT)	This enzyme is involved in the nucleotide salvage pathway, providing an alternative to nucleotide biosynthesis de novo in humans.	Deficiency	-	[102]
Hypoxanthine-guanine phosphoribosyltransferase (HPRT)	An enzyme that catalyses conversion of hypoxanthine to inosine monophosphate as well as guanine to guanosine monophosphate. This enzyme plays a crucial role in the generation of purine nucleotides by the purine salvage pathway.	Deficiency	-	[103]
Xanthine oxidase (XO)	This enzyme is involved in the generation of ROS. It catalyses the oxidation of hypoxanthine to xanthine and can further catalyse the oxidation of xanthine to uric acid.	Deficiency	-	[104]
Phosphoribosylpyrophosphate synthetase (PRPS)	An enzyme that catalyses the synthesis of phosphoribosyl pyrophosphate (PRPP), an intermediate in nucleotide metabolism and the biosynthesis of the histidine and tryptophan.	Increased activity	-	[105]
Uric acid	A product of the metabolic breakdown of purine nucleotides, and it is a normal component of urine.	Low level	Plasma	[103]
Oxy-purines	Oxygenated forms of purines.	Increased level	Plasma	[103]
Xanthine and Hypoxanthine	Xanthine is a purine base and generated on the pathway of purine degradation. It is formed from oxidation of hypoxanthine by xanthine oxidoreductase. Hypoxanthine is a spontaneous deamination product of adenine.	Increased concentrations	Plasma, urine	[103]

## Data Availability

Not applicable.
